# Identification of potential blood biomarkers for early diagnosis of Alzheimer’s disease through immune landscape analysis

**DOI:** 10.1038/s41514-022-00096-9

**Published:** 2022-11-04

**Authors:** Daichi Shigemizu, Shintaro Akiyama, Risa Mitsumori, Shumpei Niida, Kouichi Ozaki

**Affiliations:** 1 Medical Genome Center, Research Institute, National Center for Geriatrics and Gerontology, Aichi, 474-8511 Japan; 2 RIKEN Center for Integrative Medical Sciences, Yokohama, 230-0045 Japan; 3 Core Facility Administration, Research Institute, National Center for Geriatrics and Gerontology, Aichi, 474-8511 Japan

**Keywords:** Neural ageing, Biomarkers, Risk factors, Alzheimer's disease

## Abstract

Mild cognitive impairment (MCI) is a clinical precursor of Alzheimer’s disease (AD). Recent genetic studies have reported on associations between AD risk genes and immunity. Here, we obtained samples and data from 317 AD, 432 MCI, and 107 cognitively normal (CN) subjects and investigated immune-cell type composition and immune clonal diversity of T-cell receptor (TRA, TRB, TRG, and TRD) and B-cell receptor (IGH, IGK, and IGL) repertoires through bulk RNA sequencing. We found the proportions of plasma cells, γδ T cells, neutrophils, and B cells were significantly different and the diversities of IGH, IGK, and TRA were significantly small with AD progression. We then identified a differentially expressed gene, *WDR37*, in terms of risk of MCI-to-AD conversion. Our prognosis prediction model using the potential blood-based biomarkers for early AD diagnosis, which combined two immune repertoires (IGK and TRA), *WDR37*, and clinical information, successfully classified MCI patients into two groups, low and high, in terms of risk of MCI-to-AD conversion (log-rank test *P* = 2.57e-3). It achieved a concordance index of 0.694 in a discovery cohort and of 0.643 in an independent validation cohort. We believe that further investigation, using larger sample sizes, will lead to practical clinical use in the near future.

## Introduction

Mild cognitive impairment (MCI) is an intermediate stage of cognitive impairment between normal aging and dementia and is associated with an increased risk of developing clinically probable Alzheimer’s disease (AD)^1–3^. The annual conversion rate from MCI to AD is approximately 10% to 15%^4^, and most MCI patients convert to AD within 5 years from diagnosis (i.e., they are MCI converters: MCI-C)^5,6^; however, some MCI patients remain stable or convert back to being cognitively normal (CN) (i.e., they are MCI non-converters: MCI-NC)^7^. There are four drugs approved by the US Food and Drug Administration (FDA) for the management of cognitive impairment and dysfunction in symptomatic AD (three cholinesterase inhibitors: donepezil, rivastigmine, and galanthamine, and a glutamate regulator: memantine), although they can only help lessen symptoms, such as memory loss and confusion^8^. Currently the FDA has only approved aducanumab as a therapy drug for AD^9^. However, the current best strategy is to delay disease progression to reduce the number of patients who ultimately develop AD^10^. Therefore, promising biomarkers for early detection of MCI-C are urgently required because the accurate prediction of MCI-to-AD conversion would enable earlier interventions for MCI-C patients, which could lead to a reduction in MCI patients at high risk for converting to AD.

The most common multifactorial neurodegenerative disease, AD is induced by a complex interaction between genetic and environmental factors. The heritability of AD—that is, the presence of genetic risk factors—is estimated to be 60% to 80%^11^. A large number of genetic factors undoubtedly contribute to the etiopathogenesis and progression of AD, and some of them have been identified via whole-genome sequencing analyses^12,13^ and genome-wide association studies^14–16^. The AD risk genes are implicated in the immune response (*CLU, CR1, CD33, EPHA1, MS4A4E/MS4A6A, ABCA7, PTK2B, TREM2*, and *TREML2*), endocytosis (*BIN1, PICALM*, and *CD2AP*), and lipid processing (*APOE, ABCA7*, and *SORL1*)^17,18^. Thus, a large percentage of the detected AD risk genes are associated with immunity. In addition, recent clinical observations have revealed that neutrophils, essential for executing the acute inflammatory response, contribute to AD pathogenesis and cognitive impairment^19,20^. Therefore, there is consensus that the immune system is intimately involved in AD pathology, but it has yet to be established which components of the immune system actively contribute to its development.

The human adaptive immune system provides protection against an enormous variety of pathogens. The protection is mediated by receptors on the surfaces of T cells (TCRs) and B cells (BCRs). The complementarity determining region 3 (CDR3) of TCRs and BCRs is the main determinant of specificity for antigen recognition^21^. The diversity of the TCR and BCR repertoires is established during development through recombination of variable (V), diversity (D), and joining (J) genes (VDJ recombination) and gene insertion/deletion^22,23^. Previous studies have reported that the repertoire diversity plays a critical role in several diseases, including cancers^24^, autoimmune diseases^25^, and neurodegenerative diseases^26^.

High-throughput next-generation sequencing platforms enable a comprehensive assessment of the TCR and BCR repertoires, and various methodologies have been developed for the analysis of TCR and BCR repertoires from bulk RNA sequencing (RNA-seq) data (e.g., MiXCR^27^ and TRUST4^28^). Here, we performed large-scale RNA-seq transcriptome analyses using a large number of samples from persons with AD or MCI or who were CN, to detect blood-based biomarkers for early AD diagnosis. We investigated the differences in immune-cell type composition and immune clonal diversity of TCR and BCR repertoires using bulk RNA-seq data of samples with the three phenotypes (AD, MCI, and CN). We further characterized differentially expressed genes (DEGs) in the RNA-seq data between MCI-C and MCI-NC samples (i.e., prospective data). Prognosis prediction models were applied to the prospective data based on clinical information (age, sex, and *APOE* ε_4_ genotypes) and combinations of the immune-related biomarker candidates. Our final prognosis prediction model—composed of two immune repertoires (IGK and TRA), one DEG (*WDR37*), and clinical information—successfully classified the MCI patients in an independent validation cohort into two groups, high and low, in terms of risk of MCI-to-AD conversion. We believe that further investigation, using a larger sample size, will contribute to future practical clinical use in healthcare.

## Results

### RNA-sequencing data

The study included 856 samples: 317 AD, 432 MCI, and 107 CN. The average ages of the individuals from whom the AD, MCI, and CN samples were obtained were 79.3 years (SD = 5.6 years), 76.9 years (SD = 6.2 years), and 70.8 years (SD = 5.7 years), respectively, and the percentages of male subjects were 31, 42 and 51, respectively. RNA-seq analysis was performed on all samples by using the Illumina NovaSeq6000 platform. Averages of 42.9, 42.8, and 44.9 million raw read sequences were obtained from the AD, MCI, and CN samples, respectively, of which >99.5% were high quality (>Q20). After low-quality read sequences and trimmed reads with adaptor sequences were discarded, >42.3 million reads of cleaned data remained, of which >82.5% were uniquely mapped to the human reference genome (GRCh37) in the three phenotype groups (Table 1).

**Table 1:** Statistical summary of mapping results^a^.

Characteristic	AD	MCI	CN
Raw reads, n	42,851,964 ± 11,727,451	42,755,728 ± 12,595,261	44,883,385 ± 10,635,281
Q20, %	99.60 ± 0.47	99.53 ± 0.58	99.62 ± 0.44
Cleaned raw reads, n	42,378,025 ± 11,785,689	42,442,969 ± 12,532,294	44,241,452 ± 10,721,761
Unique mapped reads, %	82.54 ± 3.95	82.51 ± 3.85	82.77 ± 3.79
Multiple mapped reads, %	13.90 ± 3.68	14.05 ± 3.38	13.38 ± 3.49

*AD* Alzheimer’s disease, *MCI* mild cognitive impairment, *CN* cognitively normal.

^a^All values are mean ± SD.

### Immune-cell type composition

**Fig. 1:**
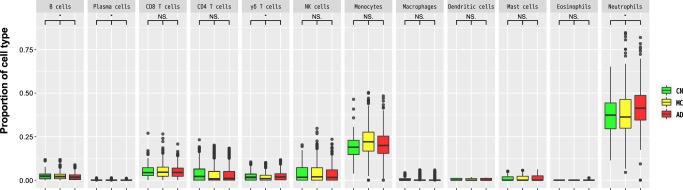
Proportions of the 12 major immune-cell types among samples from patients in each group (AD, MCI, and CN). Comparison of cell types among samples from patients with Alzheimer’s disease (AD), mild cognitive impairment (MCI), and normal cognition (CN) (*FDR < 0.05, Jonckheere–Terpstra trend test). Data are represented as box and whisker plots, depicting minimum, lower quartile (Q1), median (Q2), upper quartile (Q3), and maximum values.

We used the RNA-seq data to compare cell-type distribution among the AD, MCI, and CN samples. CIBERSORT^29^ estimated the relative proportions (as TPM) of 12 major types of immune cells (see Methods) in each sample. Statistically significant differences in cell-type proportions among the three phenotypes were assessed with the Jonckheere–Terpstra trend test. Of the 12 immune-cell types, four showed statistically significant differences among the three phenotypes at a false discovery rate (FDR) < 0.05. The proportions of plasma cells, γδ T cells, and neutrophils were significantly increased in AD progression (plasma cells, 0.026; γδ T cells, 0.034; neutrophils, 0.0024; Fig. 1) and the proportion of B cells was significantly decreased in AD progression (B cells, 0.0048; Fig. 1). We also examined the differential composition of immune-cell type among the three phenotypes by using MCP-counter^30^, but CIBERSORT detected more significant immune-cell types than MCP-counter (T cells, 0.002; B lineage, 0.016; Supplementary Fig. 1).

### Clonal diversity of T-cell receptor and B-cell receptor repertoires

**Fig. 2:**
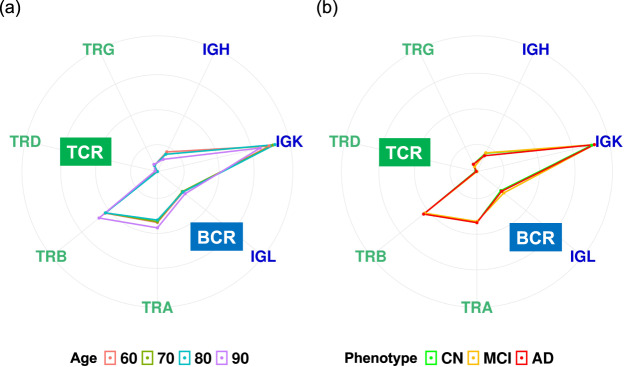
Proportion of TCR and BCR repertoires among ages and among phenotypes. Radar chart showing the proportions of the TCR and BCR repertories among ages (**a**) and among phenotypes (**b**).

**Fig. 3:**
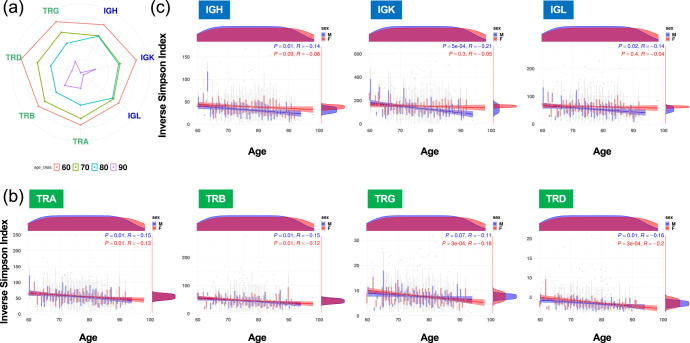
Clonal diversity of TCR and BCR repertoires among ages. (**a**) Radar chart showing a difference in clonal diversity of the TCR and BCR repertories among ages. (**b, c**) Differences in clonal diversity of the TCR (**b**) and BCR (**c**) repertoires among ages. A linear regression model was used to identify statistically significant increases or decreases in the clonal diversity of TCR and BCR repertoires among ages for each sex. The diversities of TCR repertories were significantly smaller at older ages in both sexes (**b**), whereas those of BCR repertories were significantly smaller at older ages only in male subjects at an adjusted *P* < 0.05 (**c**). Pearson correlation coefficient is represented by *R*.

**Fig. 4:**
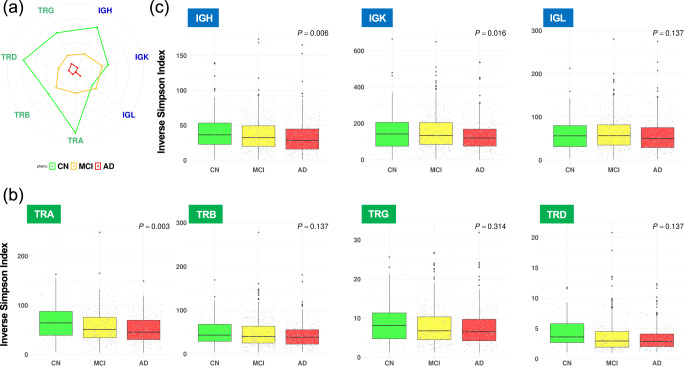
Clonal diversity of TCR and BCR repertories among phenotypes. (**a**) Radar chart showing a difference in clonal diversity of the TCR and BCR repertories among phenotypes. Difference in clonal diversity of the TCR (**b**) and BCR (**c**) repertoires among phenotypes. A linear regression model with adjustment for age and sex was used to identify statistically significant increases or decreases in the clonal diversity of TCR and BCR repertoires among phenotypes. The diversities of IGH, IGK, and TRA were statistically significantly smaller in those with AD progression at an adjusted *P* < 0.05 (**b**, **c**). Data are represented as box and whisker plots, depicting minimum, lower quartile (Q1), median (Q2), upper quartile (Q3), and maximum values (**b**, **c**).

The adaptive immune system is organized into two classes of specialized lymphocytes, T cells and B cells, which generate repertoires of T-cell receptors (TCRs) and B-cell receptors (BCRs) with sufficient diversity to recognize the universe of potential pathogens. We investigated if there were differences in proportions and diversities of TCR and BCR repertoires both at older ages and with AD progression. TRUST4 software^28^ estimated the proportions and diversities of 4 TCR (TRA, TRB, TRG, and TRD) and 3 BCR (IGH, IGK, and IGL) repertoires from bulk RNA-seq in each sample (see Methods). A linear regression model was used to identify statistically significant increases or decreases in the proportion and clonal diversity of TCR and BCR repertoires among ages and among phenotypes. Although no statistically significant differences were seen in the proportions of the TCR and BCR repertoires at older ages (Fig. 2a) or with AD progression (Fig. 2b), statistically significant decreases were observed in the diversities of their repertoires both at older ages (Fig. 3a) and with AD progression (Fig. 4a). The diversities of TCR repertories were significantly smaller at older ages in both sexes (Fig. 3b), whereas those of BCR repertories were significantly smaller at older ages only in male subjects at an FDR < 0.05 (IGH, 0.01; IGK, 5 × 10^−4^; IGL, 0.02; Fig. 3c). These results indicated that age and sex were associated with the diversities of TCR and BCR repertoires. Therefore, when assessing the associations of the diversities of their repertoires in patients with AD, we used a linear regression with adjustment for age and sex, and we found that the diversities of IGH, IGK, and TRA were statistically significantly smaller in those with AD progression at an FDR < 0.05 (IGH, 0.006; IGK, 0.016; TRA, 0.003; Fig. 4b, c).

### Identification of differentially expressed genes

**Fig. 5:**
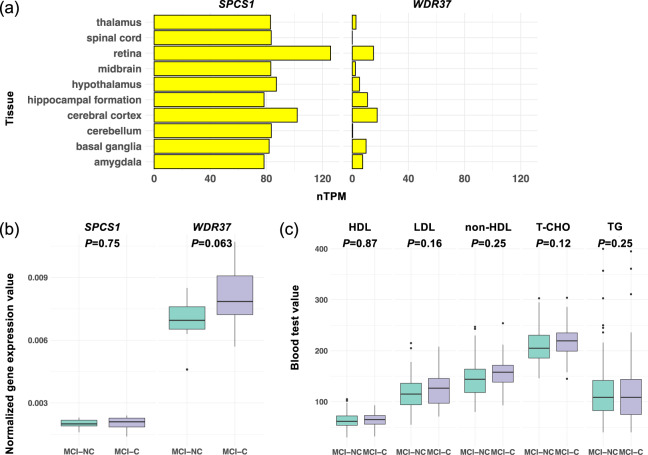
Genes differentially expressed between MCI-C and MCI-NC. (**a**) The expression of two DEGs in brain tissues was validated in the Human Protein Atlas database. The x-axis represents the consensus normalized expression (nTPM) values. (**b**) DEGs were validated by using qRT-PCR (*n* = 20; 10 MCI-C and 10 MCI-NC). (**c**) High-density lipoprotein (HDL) cholesterol, low-density lipoprotein (LDL) cholesterol, non-HDL cholesterol, total cholesterol (T-CHO), and triglycerides (TG) were measured in routine blood tests. Their differences were examined between MCI-C and MCI-NC samples (*n* = 145; 52 MCI-C and 93 MCI-NC). Welch’s *t*-test was used to identify statistically significant difference between MCI-C and MCI-NC samples in the two DEGs and five items measured in routine blood tests (**b, c**). Data are represented as box and whisker plots, depicting minimum, lower quartile (Q1), median (Q2), upper quartile (Q3), and maximum values (**b, c**).

Of the 432 MCI patients whose samples were used in RNA-seq analysis, 145 were followed for at least 6 months and up to 6 years (mean ± SD, 896.0 ± 468.2 days), during which time 52 (35.86%) converted to AD (i.e., were classified as MCI-C), and the remaining 93 MCI patients (64.14%) were classified as MCI-NC. The average ages of the MCI-C and MCI-NC subjects were 76.4 years (SD = 5.6 years) and 76.9 years (SD = 6.3 years), and the percentages of male subjects were 37 and 42, respectively (Table 2). To identify biomarker candidates for early AD diagnosis, we examined DEGs between MCI-C and MCI-NC samples from 19,702 genes with a threshold of counts per million reads mapped (CPM) > 1 in more than one-fourth of all sequenced samples using the ‘exactTest’ function in edgeR. Although no DEGs were statistically different between groups (i.e., FDR < 0.05 and fold change > 1.2), we focused on two candidate genes, with Entrez Gene IDs, showing suggestive associations (i.e., FDR < 0.2 and fold change > 1.2: *SPCS1*, FC = 1.28, *P* = 1.43 × 10^−5^, FDR = 0.11; *WDR37*, FC = 1.30, *P* = 9.56 × 10^−5^, FDR = 0.11). The expression of these candidate genes was then validated in brain tissues as well as peripheral blood mononuclear cells from the Human Protein Atlas database^31^, which provides quantitative transcriptomics at the tissue and organ level and is publicly accessible at http://www.proteinatlas.org (Fig. 5a). We also examined the differential composition of immune-cell type and clonal diversity of TCR and BCR between MCI-C and MCI-NC samples, but no statistically significant differences were observed between the groups in the composition of immune-cell types and clonal diversity of TCRs and BCRs at a false discovery rate (FDR) < 0.05 (Supplementary Table 1).

**Table 2:** Summary of sample characteristics for MCI-C and MCI-NC.

Characteristic	MCI-C	MCI-NC	All
Number of samples	52	93	145
Male, % (*n*)	37 (19)	42 (39)	40 (58)
Age, y, mean ± SD	76.4 ± 5.6	76.9 ± 6.3	76.7 ± 6.1
*APOE* ε_4_ genotypes, n (No. of patients)	0 (29), 1 (20), 2 (3)	0 (65), 1 (26), 2 (2)	0 (94), 1 (46), 2 (5)
Follow-up, mean ± SD	913.5 ± 433.4	886.3 ± 488.6	896.0 ± 468.2

*APOE* apolipoprotein E, *MCI*, mild cognitive impairment, *MCI-C* converter from MCI to Alzheimer’s disease, *MCI-NC* nonconverter from MCI to Alzheimer’s disease.

To validate our RNA-seq results, we used quantitative RT-PCR (qRT-PCR) analysis and evaluated the candidate genes detected (*SPCS1* and *WDR37*) by comparing the expression of 10 randomly selected MCI-C samples to those of 10 randomly selected MCI-NC samples. The *SPCS1* gene was not validated by the qRT-PCR (Welch’s *t*-test *P* = 0.75), whereas qRT-PCR showed a modest association for the *WDR37* gene (Welch’s *t*-test *P* = 0.063, Fig. 5b). Because *WDR37* may play a key role in cholesterol biosynthesis^32^, we investigated high-density lipoprotein (HDL) cholesterol, low-density lipoprotein cholesterol, non-HDL cholesterol, total cholesterol, and triglycerides measured in routine blood tests, but no statistically significant differences in these markers were found between MCI-C and MCI-NC samples (Fig. 5c). We further assessed associations between genetic variants on the *WDR37* gene (chr10:1102325 to 1178312) and MCI-to-AD conversion by using whole-genome sequencing data of 10 MCI-C and 17 MCI-NC samples, downloaded from the National Center for Geriatrics and Gerontology (NCGG) Biobank database. Statistically significant differences in the 271 genetic variants detected, 245 single nucleotide variants (SNVs) and 26 insertions/deletions (indels) between MCI-C and MCI-NC samples were assessed with the Fisher’s exact test, but no statistically significant associations were observed between MCI-C and MCI-NC samples in the genetic variants (Supplementary Table 2**)**.

### Prognosis prediction model construction

**Fig. 6:**
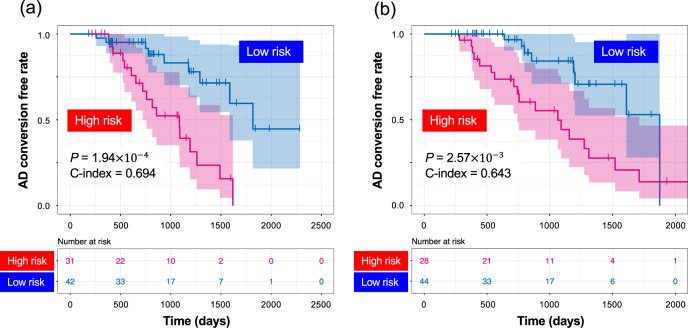
Kaplan–Meier curves of survival without conversion to AD produced by the prediction models. We calculated a prognostic index for each subject by applying three biomarkers (IGK, TRA, and *WDR37*) and clinical factors to our prognosis prediction model. (**a**) Based on the prognostic index, we divided the samples of the discovery cohort into high- (red) and low-risk (blue) groups. The optimal cutoff values were detected by using the minimum *P*-value from the log-rank test and comparing the differences in survival without MCI-to-AD conversion as determined by Kaplan-Meier curves (optimal cutoff = –0.0969, minimum *P* = 1.94 × 10^−4^). (**b**) The adjusted model was then evaluated on the validation cohort (log-rank test *P* = 2.57 × 10^−3^).

We examined blood-based biomarker candidates for early AD diagnosis through immune-cell composition, immune repertoire, and DEG analyses. In order to detect potential biomarkers from the candidates, we attempted to establish prognosis prediction models using clinical information (age, sex, and *APOE* ε_4_ genotype) and a combination of candidate markers (i.e., plasma cells, γδ T cells, neutrophils, and B cells from immune-cell compositional analyses; IGH, IGK, and TRA from the immune repertoire analyses; and *WDR37* from the DEG analyses). Prognosis prediction models were applied to a discovery cohort of 73 subjects (26 MCI-C and 47 MCI-NC). Four-fifths of the entire discovery cohort data was used for the model construction with a Cox proportional hazard model. The remaining fifth of the entire discovery cohort was used for the evaluation of the adjusted model. We used the average C-index to detect potential biomarkers. The highest C-index was observed in the fivefold cross-validation of the discovery cohort when three candidates (IGK, TRA, and *WDR37*) were used. Our final prognosis prediction model was constructed from these three potential biomarkers and clinical information using the entire discovery cohort. The adjusted model was then evaluated on a validation cohort of 72 subjects (26 MCI-C and 46 MCI-NC), which was completely independent from the discovery cohort. Our final model achieved a C-index of 0.694 in the discovery cohort and of 0.643 in the validation cohort (Fig. 6).

We further calculated a prognostic index assigned to each patient by applying the three potential biomarkers and clinical information to our prognosis prediction model. Based on the prognostic index, we divided samples of the discovery cohort into high- and low-risk groups. The optimal cutoff value was detected by using the minimum log-rank trend test *P*-value and comparing the differences in MCI-to-AD conversion–free survival as determined by Kaplan-Meier curves (optimal cutoff = -0.0969, minimum *P* = 1.94 × 10^−4^, Fig. 6a). This adjusted model was then able to successfully classify MCI patients in the validation cohort into low and high groups in terms of risk of MCI-to-AD conversion (log-rank test *P* = 2.57 × 10^−3^, Fig. 6b).

## Discussion

Although blood-based biomarkers for early diagnosis have been examined in many diseases, including AD^33–35^, there have been no robust and reliable blood biomarkers discovered that are used in routine clinical practice for AD so far. As a powerful approach to detect blood-based biomarkers, next-generation RNA-seq in human peripheral blood mononuclear cells allows a comprehensive analysis of the entire transcriptome, but many of the previous studies used only a small number of samples, especially for AD.

Most MCI patients convert to AD within 5 years of diagnosis (MCI-C)^5,6^; however, some MCI patients remain stable or convert back to being cognitively normal (MCI-NC)^7^. If accurate biomarkers that can classify MCI patients into low and high risk of MCI-to-AD conversion exist, it would enable early and targeted interventions for MCI-C patients, which could lead to a reduction in MCI patients at high risk for converting to AD. To detect potential blood-based biomarkers that could be used for early AD diagnosis, we performed comprehensive RNA-seq analysis with a large number of samples. We investigated the differences in immune-cell type composition and clonal diversity of TCR and BCR repertoires through bulk RNA-seq data from patients with AD, MCI, and CN. Of 12 major immune-cell types, four (B cells, plasma cells, γδ T cells, and neutrophils) had a significantly higher or lower cell-type proportion among the three phenotypes. It has been reported that neutrophils contribute to AD pathogenesis and cognitive impairment^19,20^, and the proportion of neutrophils might have the potential to be a blood biomarker of early AD diagnosis. However, the proportions of the remaining three cell types were very low in all samples, making it difficult to determine whether they were truly associated with the AD progression.

Age has been associated with a decrease in diversity of the TCR and BCR repertoires^36,37^, and our RNA-seq data analyses confirmed this. However, our data further showed that the diversity of BCR repertories was significantly lower at older ages only in males, whereas that of TCR repertoires showed statistically significant decreases at older ages in both sexes. In addition, we found that the diversity of IGH, IGK, and TRA was significantly lower in those diagnosed with AD, and a DEG (*WDR37*) showed a suggestive difference between MCI-C and MCI-NC samples. These results support the idea that the immune system could be used effectively for early prediction of AD diagnosis.

To identify potential biomarkers from the candidate immune-related biomarkers, we attempted to establish prognosis prediction models by using clinical information and a combination of the candidates described above. Our final prognosis prediction model achieved the highest C-index of 0.643 in the validation cohort (completely independent from the discovery cohort) when three candidates (IGK, TRA, and *WDR37*) were used.

Our study provides new information on the changes in BCR diversity associated with AD. Xu et al. previously reported that the diversity of TCR repertoires was significantly lower in samples from AD patients than in samples from CN individuals, as analyzed with single-cell RNA-seq^26^. The remaining potential biomarker, the *WDR37* gene, encodes a member of the WD40-repeat protein family and is broadly expressed in neurons of adult brains. Recent studies have shown that *WDR37* might underlie a syndromic neurological disorder^38^. Reis et al. have reported that *WDR37* may play a key role in cholesterol biosynthesis^32^, and pervasive abnormality in this system is associated with AD^39^. These results provide compelling evidence that our findings could be potential biomarkers for early detection of MCI-to-AD conversion.

We proposed an MCI-to-AD conversion prediction model based on a Cox proportional hazard method through immune landscape analysis. Although our prediction model using only biomarkers identified in this study might be insufficient to early diagnosis of AD, our findings can serve as potential biomarkers for predicting disease prognosis. In previous studies, we reported that 24 miR-eQTLs (the relationship between SNPs and miRNA expression) and three clinical factors (age, sex, and *APOE4* alleles) successfully classified MCI patients into low and high risk of MCI-to-AD conversion^13^. Moreover, recent survival analyses have showed that plasma phosphorylated tau (i.e. P-tau181 and P-tau217) and neurofilament light (NfL) could be effective biomarkers for separating MCI patients who converted to AD from those that did not^40–42^, though the AUCs were modest (P-tau181 = 0.77, NfL = 0.62)^43^. Therefore, we believe that omics analyses, using additional data including genetic variations (single nucleotide polymorphisms: SNPs, and insertions and deletions: indels) and mRNA and miRNA expressions, plasma P-tau and NfL, as well as immune-cell type composition and immune clonal diversity detected in this immune landscape analysis, will contribute to further improvement of the prognosis prediction model. As these biomarkers might also enable prediction of not only MCI-to-AD conversion, but also Amyloid/Tau/Neurodegeneration (ATN) pathology group, proposed as a means of evidencing the biological state of AD, further investigation using a larger number of samples will be required.

The main limitation of the current analyses is that it is difficult to collect many MCI-C and MCI-NC, so that our prediction model based on immune repertoires was constructed using a limited sample size. In the future, we will perform further investigations with a larger sample size and will validate the effectiveness of this classifier.

Our final prediction model based on immune repertoires successfully classified MCI patients as having low or high risk of MCI-to-AD conversion and achieved a high C-index on an independent validation cohort. Our findings showed that changes in the immune system, and specifically the immune repertoires associated with AD, could contribute to early prediction of conversion to AD. Accurate prediction of MCI-to-AD conversion would enable earlier intervention for MCI patients at high risk, potentially reducing conversion to AD.

## Methods

### Ethics approval and consent to participate

This study protocol was approved by the ethics committee of the NCGG and was done following the guidelines from the Helsinki Declaration. The design and performance of the current study involving human subjects were clearly described in a research protocol. All participation in the Biobank is voluntary, and all donors completed informed consent in writing before registering with the NCGG Biobank, and no direct or indirect identifiers have been used in reporting this manuscript.

### Sample collection

We obtained 856 blood samples with measured mRNA expression and their associated clinical data from the NCGG Biobank. Of the samples, 317 were from patients with AD, 432 were from patients with MCI, and 107 were from CN donors. Of the 432 patients with MCI, 145 were followed for more than half a year, and 52 of these 145 patients converted to AD (i.e., were MCI-C), and the remaining 93 patients remained stable with MCI (i.e., were MCI-NC). The patients from whom the AD and MCI samples were obtained were diagnosed with probable or possible AD by using the criteria of the National Institute on Aging Alzheimer’s Association workgroups^1,2^. Only samples from patients with probable AD were used as AD samples in this study. The CN samples were obtained from patients who had subjective cognitive complaints but normal cognition on a neuropsychological assessment. The diagnosis of all samples was conducted based on medical history, physical examination and diagnostic tests, neurological examination, neuropsychological tests and brain imaging with magnetic resonance imaging or computerized tomography by experts including neurologists, psychiatrists, geriatricians or a neurosurgeon, all of which are experts in dementia and familiar with its diagnostic criteria. Comprehensive neuropsychological tests included Mini-Mental State Examination (MMSE), Alzheimer’s Disease Assessment Scale Cognitive Component Japanese version, Logical Memory I and II from the Wechsler Memory Scale–Revised, frontal assessment battery, Raven’s colored progressive matrices and Geriatric Depression Scale. All samples were from men and women who were ≥60 years old at the time of testing. All CN samples had a MMSE score of >23.

### cDNA library preparation and RNA sequencing

Buffy coat samples were isolated from whole blood following the standard operating procedure of the NCGG Biobank^44^. Only high-quality samples with an RNA Integrity Number (RIN) ≥ 6.0 were used to construct the sequencing library. Sequencing libraries were prepared with 500 μg of total RNA for each sample by using Illumina TruSeq Stranded Total RNA with Ribo-Zero Globin and IDT for Illumina TruSeq UD Indexes in accordance with the manufacturer’s instructions (Illumina, San Diego, CA). The libraries were subsequently sequenced by using the Illumina NovaSeq6000 platform with paired-end reads of 151 bp in accordance with the manufacturer’s instructions.

### RNA-sequencing data analysis

All RNA-seq data were downloaded from the NCGG Biobank database. The quality of the read sequences (fastq files) was assessed with FastQC (ver. 0.11.7) and Cutadapt (ver. 1.16)^20^. The remaining clean, sequenced reads were mapped to the human reference genome (GRCh37) with STAR^45^ (ver. 2.5.2b). Read counts for each gene were calculated with the featureCounts program^46^ from the subread package (ver. 1.6.6) to generate expression levels. The read counts from each sample were combined into a count file, on which differential expression analysis was performed with edgeR^47^ (ver. 3.18.1). The ‘caclNormFactors’ function in edgeR was used to obtain TMM (trimmed mean of M-values) normalization factors to account for library sizes. The ‘exactTest’ function in edgeR was applied to obtain DEGs between MCI-C and MCI-NC samples.

### Proportion of immune-cell types

Cell-type quantification methods can be conceptually distinguished into deconvolution-based approaches and marker-gene-based approaches. We used CIBERSORT^29^ from the deconvolution-based approaches and MCP-counter^30^ from the marker-gene-based approaches. After we used STAR^45^ to align the RNA-seq reads to the human reference genome, quantification in transcripts per million (TPM) was performed with RSEM^48^ (ver. 1.3.0). The TPMs were suitable for use with the CIBERSORT (ver. 1.0.1) and MCP-counter (ver. 1.2.0). CIBERSORT estimated the proportions of 22 immune-cell types, and we further categorized the 22 cell types into 12 major cell types^20^ by summing the proportions. MCP-counter allowed robust quantification of the absolute abundance of 10 immune and stromal cell populations.

### Detection of immune receptor repertoires

Immune receptor repertoires in T cells and B cells from RNA-seq data were detected using TRUST4 software^28^ (v1.0.5), in which the inferred CDR3 clonotypes included αβ/γδ TCRs (TRA, TRB, TRG, and TRD) and BCRs (IGH, IGK, and IGL). The clonal diversity of TCRs and BCRs was estimated by using an inverse Simpson index and was calculated by using VDJtools^49^ (v1.2.1). A linear regression model was used to identify statistically significant increases or decreases in the proportion and clonal diversity of TCR and BCR repertoires among ages and among phenotypes.

### Prognosis prediction model construction

All data were strictly separated into a discovery cohort and a validation cohort. Four-fifths of the entire discovery cohort was used to construct a prognosis prediction model based on a Cox proportional hazard model by using a combination of candidate biomarkers and clinical information (age, sex, and *APOE* ε_4_ genotypes). The adjusted model was then evaluated using the remaining fifth of the discovery cohort. This process was repeated five times (5-fold cross validation). On the basis of the average concordance index (C-index), we determined the optimal combination of candidate biomarkers for model construction. The final model was constructed with the entire discovery cohort.

Using a combination of candidate biomarkers and clinical information, we calculated a prognostic index for each sample using the discovery cohort. We classified the samples into two groups (high and low risk) according to an optimal cutoff value of the prognostic index^50^. The optimal cutoff value was defined as the minimum log-rank trend test *P*-value when differences between high- and low-risk groups were compared in the discovery cohort. The optimal cutoff value was used for the validation of our prognosis prediction model. Kaplan–Meier curves were constructed to illustrate differences in MCI-to-AD conversion–free survival. The log-rank test was used to compare the different conditions. A *P*-value of 0.05 or less was considered statistically significant.

### Verification of quantitative RT-PCR assay

cDNA was synthesized by using a PrimeScript II 1st Strand cDNA Synthesis Kit (Takara Bio, Shiga, Japan). qRT-PCR analysis was performed by using TB Green Premix Ex Taq II (Takara Bio, Shiga, Japan) and the Quantstudio7 Flex Real-Time PCR System (Thermo Fisher, Waltham, MA). The following commercially available PCR primers (forward and reverse, 5ʹ to 3ʹ) were used for gene expression analysis: *SPCS1* (CCAGGCTGCTGACACTTCCT, GAGCATTAGGTGGTTTTAGCTCTTATCTG), and *WDR37* (CTGCATCAGCCGATCACACG, CGTATCTCCAGATATGAGCAGTCTG). Human beta-2-microglobulin (*hB2M*) was preselected as a reference gene for normalization of target gene expression levels. Gene expression levels from qRT-PCR were calculated relative to *hB2M* by using the semiquantitative method^51^. Gene expression levels were obtained for 10 MCI-C and 10 MCI-NC randomly selected samples. This experiment was independently performed three times for each gene.

### Reporting summary

Further information on research design is available in the Nature Research Reporting Summary linked to this article.

## Supplementary information

Supplementary Information

Reporting Summary

## Data Availability

The data that support the findings of this study are available from NBDC (National Bioscience Database Center) website under controlled access (https://humandbs.biosciencedbc.jp/en/). The NBDC number is hum0215, and the JGA (Japanese Genotype-phenotype Archive) accession number is JGAS000532.

## Code availability

We used open source program languages R (version 3.4.3) and Ruby (version 2.4.0) to analyze data and create plots. Code is available upon request from the corresponding authors.
