# Supplementary material for: Identification of potential blood biomarkers for early diagnosis of Alzheimer’s disease through immune landscape analysis
Source: NPJ Aging. 2022 Nov 4;8(1):15. doi: 10.1038/s41514-022-00096-9 (PMC9636153; doi:10.1038/s41514-022-00096-9)
Supplement: Supplementary file 1 — Supplementary Information [file 41514_2022_96_MOESM1_ESM.pdf]

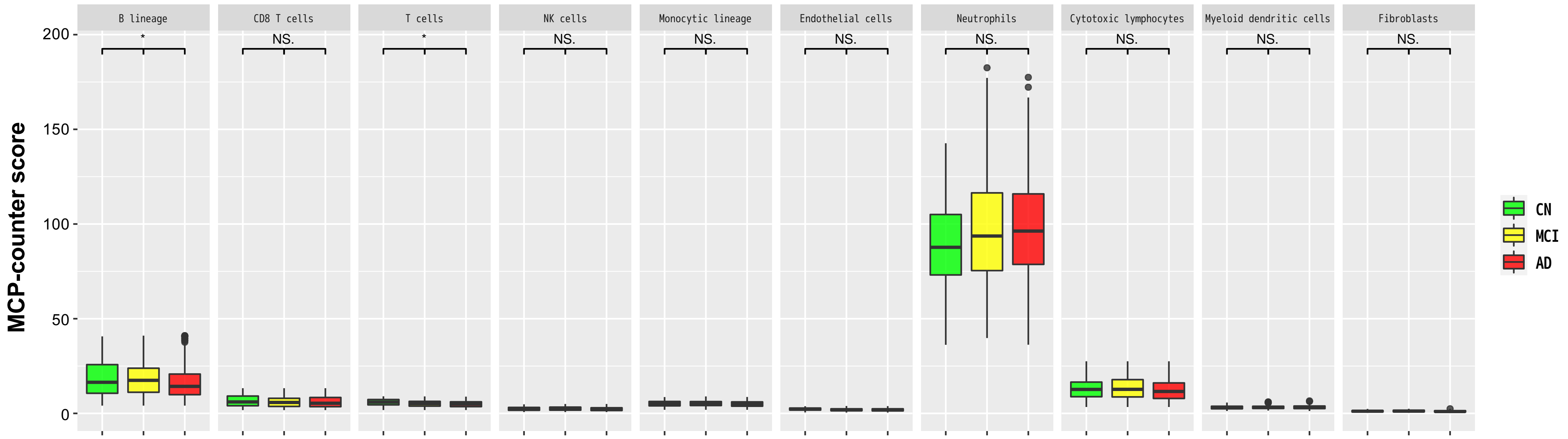

Supplementary Figure 1. Robust quantification of the absolute abundance of 10 immune and stromal cell populations.

Comparison of cell populations among samples from patients with Alzheimer's disease (AD), mild cognitive impairment (MCI), and normal cognition (CN) (*FDR < 0.05, Jonckheere–Terpstra trend test). Data are represented as box and whisker plots, depicting minimum, lower quartile (Q1), median (Q2), upper quartile (Q3), and maximum values.

Supplementary Table 1. Dfferential composition of immune-cell types and clonal diversity of TCRs and BCRs between MCI-C and MCI-NC samples

Tool	comparison	type	*P**	FDR
CIBERSORT	immune-cell	B cells	0.668	1.000
		Plasma cells	0.686	1.000
		CD8 T cells	0.262	1.000
		CD4 T cells	0.626	1.000
		γδ T cells	0.111	1.000
		NK cells	0.755	1.000
		Monocytes	0.262	1.000
		Macrophages	0.672	1.000
		Dendritic cells	0.986	1.000
		Mast cells	0.793	1.000
		Eosinophils	1.000	1.000
		Neutrophils	0.347	1.000
MCP-counter	immune-cell	B lineage	0.998	1.000
		CD8 T cells	0.449	1.000
		T cells	0.139	1.000
		NK cells	0.862	1.000
		Monocytic lineage	0.994	1.000
		Endothelial cells	0.261	1.000
		Neutrophils	0.090	0.898

		Cytotoxic lymphocytes	0.582	1.000
		Myeloid dendritic cells	0.876	1.000
		Fibroblasts	0.336	1.000
TRUST4	clonal diversity	IGH	0.381	0.781
		IGK	0.139	0.781
		IGL	0.747	0.781
		TRA	0.307	0.781
		TRB	0.649	0.781
		TRD	0.741	0.781
		TRG	0.781	0.781

*: Statistically significant differences in cell-type proportions between MCI-C and MCI-NC samples were assessed with the Jonckheere–Terpstra trend test. A linear regression model with adjustment for age and sex was used to identify statistically significant increases or decreases in the clonal diversity of TCR and BCR repertoires between MCI-C and MCI-NC samples.

Supplementary Table 2. The association between genetic variants on *WDR37* and MCI-to-AD conversion

chromosome	position	SNP ID	variant	A1 allele	A2 allele	MCI-C (A1/A1A2/A2)	MCI-NC (A1/A1A2/A2)	*P**
10	1102613	rs2306403	SNV	A	C	0/2/8	0/6/11	0.6655
10	1102710	rs11250244	SNV	A	C	0/3/7	0/6/11	1
10	1102796	rs35656218	SNV	C	G	0/5/5	3/6/8	0.4714
10	1102868	rs10047382	SNV	C	T	0/1/4	0/0/2	1
10	1103058	rs10047269	SNV	A	G	0/1/4	0/0/2	1
10	1103095	rs10047383	SNV	G	A	0/1/4	0/0/2	1
10	1103253	rs147745963	INDEL	G	GGGCGCTGGAGGGCCCGGGGC	0/1/4	0/0/2	1
10	1103265	10:1103265	INDEL	GCCC	G	1/1/8	0/1/16	0.4575
10	1103590	rs35657988	SNV	G	A	0/1/9	0/1/16	1
10	1104027	10:1104027	INDEL	G	GGAGTTA	0/6/4	4/5/8	0.177
10	1104257	rs12268949	SNV	G	A	0/1/4	0/0/2	1
10	1105019	rs7101191	SNV	C	A	0/2/8	0/6/11	0.6655
10	1105171	rs75431717	SNV	A	C	0/1/9	0/1/16	1
10	1105322	rs72760934	SNV	A	G	0/1/9	0/1/16	1
10	1105473	rs7071670	SNV	A	T	0/1/4	0/0/2	1
10	1105967	10:1105967	SNV	T	C	0/1/9	0/0/17	0.3704
10	1107341	rs7093915	SNV	T	C	0/1/4	0/0/2	1
10	1107422	rs72760935	SNV	G	A	0/1/9	0/1/15	1

10	1107476	rs190535952	SNV	T	C	0/1/7	0/0/16	0.3333
10	1107697	rs7093383	SNV	G	T	0/1/9	0/5/12	0.3625
10	1107888	rs181489269	SNV	G	A	0/0/10	0/1/16	1
10	1108544	rs10903359	SNV	G	T	0/3/7	0/7/10	0.6919
10	1109356	rs201435385	SNV	T	C	0/1/5	0/0/16	0.2727
10	1110231	rs7100114	SNV	G	A	0/1/4	0/0/2	1
10	1110369	rs147957107	SNV	T	C	0/1/9	0/0/17	0.3704
10	1110576	rs11250245	SNV	A	C	0/1/0	0/0/0	1
10	1112149	rs11815047	SNV	C	G	0/2/8	0/1/16	0.535
10	1112266	rs7079564	SNV	C	T	0/7/3	1/5/11	0.1071
10	1112277	10:1112277	SNV	T	C	0/0/5	0/1/14	1
10	1112278	rs11250246	SNV	A	G	0/2/8	1/6/10	0.6347
10	1112280	rs201266334	SNV	T	A	0/0/10	0/1/16	1
10	1112291	rs188637100	SNV	C	T	0/0/10	0/1/16	1
10	1112298	10:1112298	SNV	T	C	0/0/8	0/1/16	1
10	1112300	rs116959157	SNV	C	T	0/0/10	0/1/16	1
10	1112303	rs201001931	SNV	G	A	0/0/10	0/1/16	1
10	1112309	rs145416272	SNV	G	A	0/0/10	0/1/16	1
10	1112357	rs187039752	SNV	A	G	0/1/7	0/1/15	1
10	1112421	rs10903360	SNV	A	G	0/6/4	3/7/7	0.4009
10	1113305	rs2127605	SNV	G	C	0/6/4	4/5/8	0.177
10	1114339	rs10444196	SNV	G	A	0/6/4	3/5/8	0.23

10	1116042	rs193178104	SNV	G	A	0/0/9	1/0/16	1
10	1116294	rs4519019	SNV	G	A	0/6/4	4/5/8	0.177
10	1116671	rs2387217	SNV	G	A	0/2/8	0/6/11	0.6655
10	1117459	rs80141980	SNV	T	G	0/1/8	0/5/12	0.3798
10	1117514	rs11250250	SNV	C	T	0/2/8	0/6/11	0.6655
10	1117764	rs7083203	SNV	T	C	0/4/6	1/5/11	0.8064
10	1118301	rs191792429	SNV	T	C	0/0/10	0/2/15	0.5157
10	1119203	rs10508203	SNV	G	A	0/4/6	1/5/11	0.8064
10	1119848	10:1119848	SNV	A	G	0/0/5	0/1/14	1
10	1120061	rs141413817	SNV	C	A	0/0/10	0/1/16	1
10	1120097	10:1120097	SNV	G	A	0/1/9	0/0/17	0.3704
10	1120236	rs41299206	SNV	C	T	0/1/4	0/0/2	1
10	1120394	10:1120394	SNV	A	G	0/0/5	0/1/14	1
10	1120600	rs7070991	SNV	A	G	0/1/9	0/1/16	1
10	1120884	rs7072276	SNV	T	C	0/4/6	1/5/11	0.8064
10	1121381	rs55701841	SNV	C	T	0/5/3	4/5/7	0.2709
10	1121814	rs10219038	SNV	A	G	0/1/4	0/0/2	1
10	1121939	rs76732489	SNV	T	C	0/1/4	0/0/2	1
10	1122895	10:1122895	SNV	G	A	0/0/9	0/1/14	1
10	1123453	rs7085063	SNV	T	G	0/1/9	0/5/12	0.3625
10	1123937	rs144312898	SNV	A	T	0/0/9	0/1/16	1
10	1124186	rs143298688	SNV	C	T	0/0/10	0/1/16	1

10	1124531	rs7091030	SNV	T	C	0/1/9	0/1/16	1
10	1124939	rs11250252	SNV	C	G	0/1/4	0/0/2	1
10	1125147	rs10794715	SNV	C	T	0/6/4	4/5/8	0.177
10	1125160	rs149683159	SNV	A	G	0/1/9	1/1/15	1
10	1125327	rs202070825	INDEL	G	GAAT	0/1/9	1/1/15	1
10	1125344	rs147803324	SNV	A	G	0/0/10	0/1/16	1
10	1125397	rs146023579	INDEL	GACCCTGATAGCAGTGTCTTCCAC	G	0/1/9	1/5/11	0.4544
10	1125769	rs11250253	SNV	C	A	0/2/8	0/6/11	0.6655
10	1126293	rs7087381	SNV	C	T	0/6/4	4/5/8	0.177
10	1126619	rs7084283	SNV	G	A	0/6/4	4/5/8	0.177
10	1126866	rs74120451	SNV	C	T	0/1/4	0/0/2	1
10	1126904	rs7101036	SNV	A	C	0/1/9	0/1/16	1
10	1126969	rs7101059	SNV	G	C	0/1/9	0/1/16	1
10	1127151	rs57019561	INDEL	TG	T	0/1/9	0/1/16	1
10	1127230	rs17155873	SNV	T	G	0/1/9	0/1/16	1
10	1128103	rs370140003	SNV	T	C	0/1/9	0/1/16	1
10	1128165	rs78493356	SNV	G	A	0/1/9	0/0/17	0.3704
10	1128216	rs7074721	SNV	G	A	0/6/4	4/5/8	0.177
10	1128238	rs60503326	SNV	A	G	0/1/9	0/1/16	1
10	1128663	rs200855697	INDEL	C	CAT	0/1/8	0/1/11	1
10	1128812	rs11819230	SNV	G	A	0/1/4	0/0/2	1

10	1128950	rs11250254	SNV	A	G	0/2/8	0/6/11	0.6655
10	1129527	rs7080072	SNV	T	G	0/1/9	0/5/12	0.3625
10	1129680	rs148186629	SNV	T	C	0/0/10	1/0/16	1
10	1129814	rs7080500	SNV	A	G	0/3/7	0/6/11	1
10	1130127	rs7093837	SNV	T	C	0/1/9	0/1/16	1
10	1130756	rs10903361	SNV	A	G	0/6/4	4/5/8	0.177
10	1131050	10:1131050	SNV	A	G	0/0/8	1/0/15	1
10	1131177	rs59784176	SNV	T	G	0/1/9	0/1/16	1
10	1131270	rs57295313	INDEL	TC	T	0/1/9	0/1/16	1
10	1131325	rs117348574	SNV	G	C	0/1/9	0/2/15	1
10	1131398	rs191077947	SNV	A	G	0/1/9	0/0/17	0.3704
10	1132132	rs7914308	SNV	C	T	0/1/9	0/1/16	1
10	1132848	rs7069496	SNV	C	G	0/1/9	0/1/16	1
10	1132852	rs2279256	SNV	A	G	0/1/9	0/1/16	1
10	1133004	rs137942892	SNV	T	C	0/1/9	0/1/16	1
10	1133161	rs117064546	SNV	T	C	0/2/8	0/2/15	0.6125
10	1133435	10:1133435	SNV	T	C	0/1/4	0/0/15	0.25
10	1133628	rs1871625	SNV	T	A	0/4/6	1/5/11	0.8064
10	1133974	rs2101050	SNV	C	T	0/6/4	4/5/8	0.177
10	1134335	rs11250256	SNV	G	A	0/6/4	4/5/8	0.177
10	1135221	rs4880473	SNV	T	C	0/2/8	1/4/12	1
10	1135248	rs4880474	SNV	T	C	0/1/9	0/5/12	0.3625

10	1135520	10:1135520	SNV	G	A	0/1/9	0/0/17	0.3704
10	1135642	rs201335374	INDEL	C	CGTGCTCA CCCGCAAT TCAACCTT CCT	0/2/8	0/6/11	0.6655
10	1135757	rs34766854	SNV	T	C	0/6/4	4/6/7	0.2837
10	1136330	rs7076083	SNV	C	T	0/6/4	4/5/8	0.177
10	1136357	rs182614153	SNV	T	C	0/0/10	0/1/16	1
10	1136557	rs59961913	INDEL	T	TC	0/2/8	0/5/12	0.6784
10	1137009	rs149466850	INDEL	C	CAG	0/0/8	0/2/15	1
10	1137266	rs10903362	SNV	T	G	0/6/4	4/5/8	0.177
10	1137842	rs11250258	SNV	T	C	0/6/4	4/5/8	0.177
10	1137992	10:1137992	SNV	T	C	0/0/5	0/1/14	1
10	1138044	10:1138044	SNV	C	T	0/0/6	0/2/13	1
10	1138168	rs10903365	SNV	C	T	0/2/3	1/1/0	0.5714
10	1138370	10:1138370	SNV	A	G	0/0/10	0/1/16	1
10	1138534	rs67388830	SNV	A	G	0/6/4	4/5/8	0.177
10	1138620	rs7921047	SNV	A	G	0/1/9	0/1/16	1
10	1139183	rs10159718	SNV	G	A	0/6/4	4/5/8	0.177
10	1139469	10:1139469	SNV	C	G	0/0/8	0/2/14	0.5362
10	1139628	rs142664072	SNV	G	A	0/0/10	0/1/16	1
10	1139825	rs10903366	SNV	G	A	0/6/4	4/5/8	0.177
10	1140864	rs183185700	SNV	A	G	0/0/10	0/1/16	1

10	1140962	10:1140962	INDEL	C	CAT	0/1/9	0/1/15	1
10	1141102	rs10903367	SNV	G	A	0/2/3	1/1/0	0.5714
10	1141183	rs12569814	SNV	A	G	0/6/4	3/5/7	0.2751
10	1141666	rs56201968	SNV	G	A	0/1/9	0/1/16	1
10	1141749	rs3816735	SNV	A	G	0/6/4	4/5/8	0.177
10	1142424	rs10794717	SNV	G	A	0/4/6	0/8/9	1
10	1142571	rs36062650	SNV	C	T	0/1/9	0/1/16	1
10	1142595	rs72760942	SNV	A	G	0/1/9	0/2/15	1
10	1142750	rs10794718	SNV	T	C	0/5/5	4/5/8	0.2826
10	1142860	rs59245488	SNV	T	A	0/1/9	0/1/16	1
10	1142898	rs180728743	SNV	C	G	0/0/10	0/2/15	0.5157
10	1143212	rs67615426	SNV	A	G	0/1/9	0/1/16	1
10	1143250	rs145542675	SNV	T	C	0/1/9	0/1/16	1
10	1143254	rs116887570	SNV	A	G	0/0/10	0/1/16	1
10	1143827	rs12784270	SNV	C	T	0/6/4	4/6/7	0.2837
10	1144049	rs7067536	SNV	T	C	0/1/9	0/1/16	1
10	1144254	rs117014471	SNV	G	C	0/3/7	1/3/13	0.776
10	1144594	10:1144594	INDEL	C	CT	0/0/5	0/1/14	1
10	1144630	rs3793774	SNV	G	A	1/5/4	4/6/7	0.8709
10	1145182	rs17155886	SNV	G	A	0/2/8	0/1/16	0.535
10	1145284	rs34964132	SNV	A	C	0/5/5	0/7/10	0.7063
10	1145473	rs11250260	SNV	A	T	0/6/4	4/6/7	0.2837

10	1145635	rs11250261	SNV	A	G	0/1/9	0/7/10	0.1895
10	1145697	rs144775512	SNV	C	T	0/1/9	0/0/16	0.3846
10	1145707	rs74117831	SNV	C	A	0/2/7	0/1/15	0.5304
10	1145768	10:1145768	SNV	A	G	0/1/4	0/0/15	0.25
10	1145839	10:1145839	INDEL	A	AGTGGTGGAGAGGTGAGGAGGGTGAGCACTGATGCTCAGGAAATTCAAAATGTGCACTGATGGTGGGC	0/1/9	0/7/9	0.09885
10	1146169	rs57514701	SNV	A	G	0/2/8	0/1/16	0.535
10	1146223	rs58551457	SNV	A	G	0/1/2	0/1/0	1
10	1146226	rs143886758	INDEL	G	GCACTGCA	0/6/4	4/6/7	0.2837
10	1147138	rs11250263	SNV	G	A	0/2/8	0/5/12	0.6784
10	1148157	rs11818314	SNV	C	G	0/1/4	0/0/2	1
10	1148301	10:1148301	SNV	C	T	0/1/9	0/0/16	0.3846
10	1148336	rs113148608	INDEL	T	TA	0/1/9	0/1/16	1
10	1148619	rs149876336	SNV	T	C	0/2/8	0/0/17	0.1282
10	1148672	rs10903368	SNV	G	C	0/3/7	0/7/10	0.6919
10	1149390	rs7915650	SNV	A	C	0/2/8	0/5/12	0.6784

10	1150082	10:1150082	INDEL	TGACGGCC TCATGGTTT CCCA	T	0/0/6	0/1/15	1
10	1151620	rs4880765	SNV	G	A	2/4/4	4/6/7	1
10	1151681	10:1151681	SNV	T	C	0/0/5	0/1/14	1
10	1151723	rs11250264	SNV	C	A	0/2/8	0/1/16	0.535
10	1152067	rs11250265	SNV	C	T	0/5/5	0/7/10	0.7063
10	1152480	rs57552534	SNV	A	G	0/1/9	0/1/15	1
10	1152862	rs55886714	SNV	G	T	0/3/7	0/6/11	1
10	1153222	rs7894567	SNV	G	A	0/5/5	0/7/10	0.7063
10	1153725	rs139821411	SNV	T	C	0/0/10	0/1/15	1
10	1153731	rs10903369	SNV	C	T	0/3/7	0/7/10	0.6919
10	1153749	rs2387296	SNV	G	A	0/3/7	0/7/10	0.6919
10	1153816	rs10736941	SNV	C	T	2/4/4	4/6/7	1
10	1153856	rs57242050	INDEL	C	CAT	0/3/7	0/2/15	0.3261
10	1154019	rs4880766	SNV	C	T	2/4/4	4/6/7	1
10	1154105	rs4880767	SNV	C	G	0/5/5	0/7/10	0.7063
10	1154669	rs3886474	SNV	T	C	0/1/3	0/0/1	1
10	1155393	rs74572057	SNV	A	G	0/2/8	0/0/17	0.1282
10	1155394	rs61693569	SNV	T	C	0/1/9	0/1/16	1
10	1155608	rs72635992	SNV	A	G	0/3/7	0/7/10	0.6919
10	1155767	rs11250266	SNV	T	A	0/2/8	0/5/12	0.6784
10	1156165	rs10794720	SNV	T	C	0/2/8	0/1/16	0.535

10	1156499	rs139376446	SNV	G	C	0/1/9	0/1/16	1
10	1156815	rs7922760	SNV	G	C	0/2/8	0/1/16	0.535
10	1156864	rs10903370	SNV	G	A	2/4/4	4/6/7	1
10	1156943	rs61144178	INDEL	C	CAT	0/2/8	0/1/16	0.535
10	1156994	rs72760953	SNV	C	T	0/1/9	0/1/16	1
10	1157019	rs59377495	SNV	T	C	0/2/8	0/1/16	0.535
10	1157391	rs182001224	SNV	C	G	0/1/9	0/0/17	0.3704
10	1157410	rs7088740	SNV	G	A	2/2/6	4/5/8	0.8754
10	1157936	10:1157936	SNV	A	G	0/0/10	0/1/16	1
10	1158376	rs2086382	SNV	G	A	0/3/7	0/7/10	0.6919
10	1159187	rs56004802	SNV	T	C	0/2/8	0/1/16	0.535
10	1159566	rs11250268	SNV	A	C	0/3/7	0/7/10	0.6919
10	1159648	rs72760954	SNV	T	C	0/1/9	0/1/16	1
10	1160101	rs184189632	SNV	T	C	0/1/5	0/0/16	0.2727
10	1160877	rs117921987	SNV	C	T	0/0/10	0/4/13	0.2638
10	1161120	rs10903371	SNV	T	C	2/4/4	4/6/7	1
10	1161653	rs1079390	SNV	A	C	0/2/8	0/1/16	0.535
10	1161745	rs1079387	SNV	A	G	0/1/9	0/7/10	0.1895
10	1161824	rs1079388	SNV	G	A	2/4/4	4/6/7	1
10	1161980	rs1079389	SNV	G	A	0/2/8	0/1/16	0.535
10	1162174	rs2387297	SNV	C	T	0/5/5	0/7/10	0.7063
10	1162279	rs2387298	SNV	T	C	0/1/9	0/1/15	1

10	1162776	rs117859365	SNV	T	C	0/0/10	0/1/16	1
10	1162846	rs56702647	INDEL	G	GAGAATCCTTGTCTCTTACCAGCAGAGCAGCGC	0/1/9	0/1/16	1
10	1163075	rs10751791	SNV	A	G	2/3/5	2/7/8	0.8719
10	1163427	rs2387299	SNV	G	A	0/2/8	0/1/16	0.535
10	1163598	rs2387300	SNV	A	G	0/2/8	0/0/17	0.1282
10	1163854	rs11250269	SNV	G	A	0/5/4	1/4/11	0.2839
10	1164969	10:1164969	INDEL	G	GCCATT	0/1/9	0/0/17	0.3704
10	1165425	rs74117849	SNV	G	A	0/2/8	0/1/16	0.535
10	1165777	rs74117850	SNV	C	T	0/2/8	0/1/16	0.535
10	1166128	rs79934515	SNV	T	C	0/3/7	0/2/15	0.3261
10	1166318	rs11250270	SNV	A	G	0/2/8	0/5/12	0.6784
10	1166319	rs11250271	SNV	A	G	0/2/8	0/5/12	0.6784
10	1166400	rs1871626	SNV	C	T	2/3/5	2/7/8	0.8719
10	1166600	rs59686718	SNV	A	G	0/4/6	0/5/12	0.6831
10	1166687	rs5014941	SNV	C	G	2/3/5	2/7/8	0.8719
10	1167995	10:1167995	SNV	C	G	0/1/4	0/0/15	0.25
10	1168026	rs201454691	INDEL	C	CGCATAGA	0/1/9	0/3/14	1
10	1168267	rs7899518	SNV	C	A	2/7/1	3/6/8	0.1639
10	1168503	rs376019718	SNV	T	C	0/1/9	0/1/16	1

10	1168563	rs6560711	SNV	G	A	0/2/8	0/5/12	0.6784
10	1169678	rs370071628	SNV	G	A	0/1/5	0/0/16	0.2727
10	1169907	rs72760963	SNV	A	G	0/1/9	0/1/16	1
10	1169992	rs74591529	SNV	T	C	0/1/9	0/1/16	1
10	1170304	rs7916430	SNV	G	C	0/2/8	0/6/11	0.6655
10	1170383	10:1170383	SNV	C	A	0/0/5	0/1/14	1
10	1170400	rs72760964	SNV	G	A	0/1/9	0/1/16	1
10	1171266	rs11250272	SNV	C	G	2/4/3	3/7/6	1
10	1171311	rs11250273	SNV	G	A	2/4/3	3/7/6	1
10	1171352	rs143017085	SNV	C	G	0/2/8	0/1/16	0.535
10	1171354	rs11250274	SNV	C	T	0/0/10	0/1/16	1
10	1171734	rs10903372	SNV	T	C	0/2/8	0/5/12	0.6784
10	1171822	10:1171822	SNV	T	C	0/1/4	0/0/15	0.25
10	1171823	rs11250275	SNV	A	G	0/0/10	0/1/16	1
10	1172081	rs141729568	SNV	C	G	0/0/10	0/1/16	1
10	1172094	10:1172094	SNV	C	G	0/0/10	0/1/16	1
10	1172095	10:1172095	SNV	C	T	0/0/10	0/1/16	1
10	1172172	rs111964088	SNV	C	T	0/1/7	0/0/16	0.3333
10	1172198	rs59259654	SNV	C	T	2/3/5	5/8/4	0.4721
10	1172203	rs113900916	SNV	G	A	0/5/5	3/4/10	0.2186
10	1172208	rs371089630	SNV	C	T	0/0/8	0/1/16	1
10	1172225	rs71491340	SNV	G	A	0/0/10	0/1/16	1

10	1172249	rs7101147	SNV	G	A	0/7/2	1/7/8	0.2739
10	1172271	rs7085880	SNV	G	A	4/2/4	1/9/7	0.09197
10	1172283	rs12265731	SNV	C	T	0/1/9	0/1/16	1
10	1172294	rs10794722	SNV	C	T	0/8/2	4/6/7	0.08242
10	1172308	rs182624320	SNV	C	T	0/0/10	0/1/16	1
10	1172360	rs369065820	SNV	A	G	0/1/9	0/1/16	1
10	1172589	10:1172589	SNV	G	A	0/1/9	0/0/17	0.3704
10	1172839	rs7894626	SNV	A	C	1/7/2	5/7/5	0.4435
10	1172851	rs10160136	SNV	T	C	2/5/3	1/8/8	0.5469
10	1173405	rs11356997	INDEL	C	CT	0/1/9	0/2/15	1
10	1173670	rs4880770	SNV	A	G	0/3/7	1/8/8	0.6398
10	1173672	rs4880771	SNV	T	C	0/3/7	1/8/8	0.6398
10	1173689	rs10903373	SNV	T	C	0/3/7	2/7/8	0.5835
10	1173736	10:1173736	SNV	A	G	0/0/7	0/1/14	1
10	1174843	10:1174843	INDEL	TCGGCGGTCCATGCTCGG	T	0/0/8	1/0/15	1
10	1174844	10:1174844	SNV	C	T	0/0/5	0/1/13	1
10	1174859	10:1174859	SNV	G	T	0/0/5	0/1/12	1
10	1174860	10:1174860	SNV	G	A	0/0/5	0/1/12	1
10	1174866	10:1174866	INDEL	C	CATGGGG	0/0/5	0/1/14	1
10	1175426	rs184435191	SNV	G	C	0/0/7	0/1/14	1
10	1175894	rs11250281	SNV	G	A	0/5/5	2/10/5	0.492

10	1175939	rs12359210	SNV	C	T	1/4/5	4/10/3	0.3118
10	1176813	rs149181350	INDEL	G	GC	0/1/9	0/2/15	1
10	1177236	rs76312690	SNV	T	C	0/1/9	0/2/15	1
10	1178211	rs187772252	SNV	C	T	0/1/9	0/0/17	0.3704

*: Statistically significant differences in the genetic variants between MCI-C and MCI-NC samples were assessed with the Fisher's exact test.
