# Supplementary material for: Identification of potential blood biomarkers for early diagnosis of Alzheimer’s disease through immune landscape analysis
Source: NPJ Aging. 2022 Nov 4;8(1):15. doi: 10.1038/s41514-022-00096-9 (PMC9636153; doi:10.1038/s41514-022-00096-9)
Supplement: Supplementary file 2 — Reporting Summary [file 41514_2022_96_MOESM2_ESM.pdf]

nature portfolio

Corresponding author(s): Daichi Shigemizu

Last updated by author(s): Sep 28, 2022

Reporting Summary

Nature Portfolio wishes to improve the reproducibility of the work that we publish. This form provides structure for consistency and transparency in reporting. For further information on Nature Portfolio policies, see our Editorial Policies and the Editorial Policy Checklist.

Statistics

For all statistical analyses, confirm that the following items are present in the figure legend, table legend, main text, or Methods section.

n/a	Confirmed	
☐	☒	The exact sample size (n) for each experimental group/condition, given as a discrete number and unit of measurement
☐	☒	A statement on whether measurements were taken from distinct samples or whether the same sample was measured repeatedly
☐	☒	The statistical test(s) used AND whether they are one- or two-sided<br>*Only common tests should be described solely by name; describe more complex techniques in the Methods section.*
☐	☒	A description of all covariates tested
☐	☒	A description of any assumptions or corrections, such as tests of normality and adjustment for multiple comparisons
☐	☒	A full description of the statistical parameters including central tendency (e.g. means) or other basic estimates (e.g. regression coefficient) AND variation (e.g. standard deviation) or associated estimates of uncertainty (e.g. confidence intervals)
☐	☒	For null hypothesis testing, the test statistic (e.g. F, t, r) with confidence intervals, effect sizes, degrees of freedom and P value noted<br>*Give P values as exact values whenever suitable.*
☒	☐	For Bayesian analysis, information on the choice of priors and Markov chain Monte Carlo settings
☒	☐	For hierarchical and complex designs, identification of the appropriate level for tests and full reporting of outcomes
☐	☒	Estimates of effect sizes (e.g. Cohen's d, Pearson's r), indicating how they were calculated

Our web collection on statistics for biologists contains articles on many of the points above.

Software and code

Policy information about availability of computer code

Data collection	No software was used.
Data analysis	We used open source program languages R (version 3.4.3) and Ruby (version 2.4.0) to analyze data and create plots. Code is available upon request from the corresponding authors.

For manuscripts utilizing custom algorithms or software that are central to the research but not yet described in published literature, software must be made available to editors and reviewers. We strongly encourage code deposition in a community repository (e.g. GitHub). See the Nature Portfolio guidelines for submitting code & software for further information.

Data

Policy information about availability of data

All manuscripts must include a data availability statement. This statement should provide the following information, where applicable:

- Accession codes, unique identifiers, or web links for publicly available datasets
- A description of any restrictions on data availability
- For clinical datasets or third party data, please ensure that the statement adheres to our policy

The data that support the findings of this study are available from NBDC (National Bioscience Database Center) website under controlled access (https://humandbs.biosciencedbc.jp/en/). The NBDC number is hum0215, and the JGA (Japanese Genotype-phenotype Archive) accession number is JGAS000532.

Human research participants

Policy information about studies involving human research participants and Sex and Gender in Research.

Reporting on sex and gender	We obtained 856 blood samples with measured mRNA expression and their associated clinical data from the NCGG Biobank. Of the samples, 333 were male and the remaining 523 were female.
Population characteristics	Of 856 samples, 317 were from patients with AD, 432 were from patients with MCI, and 107 were from CN donors. Of the 432 patients with MCI, 145 were followed for more than half a year, and 52 of these 145 patients converted to AD (i.e., were MCI-C), and the remaining 93 patients remained stable with MCI (i.e., were MCI-NC). All samples were from men and women who were ≥ 60 years old at the time of testing.
Recruitment	This study protocol was approved by the ethics committee of the NCGG and was done following the guidelines from the Helsinki Declaration. The design and performance of the current study involving human subjects were clearly described in a research protocol. All participation in the Biobank is voluntary.
Ethics oversight	All donors completed informed consent in writing before registering with the NCGG Biobank.

Note that full information on the approval of the study protocol must also be provided in the manuscript.

Field-specific reporting

Please select the one below that is the best fit for your research. If you are not sure, read the appropriate sections before making your selection.

☒ Life sciences ☐ Behavioural & social sciences ☐ Ecological, evolutionary & environmental sciences

For a reference copy of the document with all sections, see nature.com/documents/nr-reporting-summary-flat.pdf

Life sciences study design

All studies must disclose on these points even when the disclosure is negative.

Sample size	We obtained 856 blood samples with measured mRNA expression and their associated clinical data from the NCGG Biobank.
Data exclusions	No data were excluded from the analyses.
Replication	All attempts at experimental replication, for the data presented in this report, were successful.
Randomization	Samples were allocated into experimental groups randomly.
Blinding	Investigators were blinded to group allocation in the RNA-seq.

Reporting for specific materials, systems and methods

We require information from authors about some types of materials, experimental systems and methods used in many studies. Here, indicate whether each material, system or method listed is relevant to your study. If you are not sure if a list item applies to your research, read the appropriate section before selecting a response.

Materials & experimental systems

n/a	Involved in the study	
☒	☐	Antibodies
☒	☐	Eukaryotic cell lines
☒	☐	Palaeontology and archaeology
☒	☐	Animals and other organisms
☒	☐	Clinical data
☒	☐	Dual use research of concern

Methods

n/a	Involved in the study	
☒	☐	ChIP-seq
☒	☐	Flow cytometry
☒	☐	MRI-based neuroimaging
